# A Framework for Optimizing Co-adaptation in Body-Machine Interfaces

**DOI:** 10.3389/fnbot.2021.662181

**Published:** 2021-04-21

**Authors:** Dalia De Santis

**Affiliations:** Department of Robotics, Brain and Cognitive Sciences, Center for Human Technologies, Istituto Italiano di Tecnologia, Genova, Italy

**Keywords:** co-adaptation, human-machine interface, use-dependent learning, model-free learning, reinforcement, dimensionality reduction, subspace learning, body-machine interface

## Abstract

The operation of a human-machine interface is increasingly often referred to as a two-learners problem, where both the human and the interface independently adapt their behavior based on shared information to improve joint performance over a specific task. Drawing inspiration from the field of body-machine interfaces, we take a different perspective and propose a framework for studying co-adaptation in scenarios where the evolution of the interface is dependent on the users' behavior and that do not require task goals to be explicitly defined. Our mathematical description of co-adaptation is built upon the assumption that the interface and the user agents co-adapt toward maximizing the interaction efficiency rather than optimizing task performance. This work describes a mathematical framework for body-machine interfaces where a naïve user interacts with an adaptive interface. The interface, modeled as a linear map from a space with high dimension (the user input) to a lower dimensional feedback, acts as an adaptive “tool” whose goal is to minimize transmission loss following an unsupervised learning procedure and has no knowledge of the task being performed by the user. The user is modeled as a non-stationary multivariate Gaussian generative process that produces a sequence of actions that is either statistically independent or correlated. Dependent data is used to model the output of an action selection module concerned with achieving some unknown goal dictated by the task. The framework assumes that in parallel to this explicit objective, the user is implicitly learning a suitable but not necessarily optimal way to interact with the interface. Implicit learning is modeled as use-dependent learning modulated by a reward-based mechanism acting on the generative distribution. Through simulation, the work quantifies how the system evolves as a function of the learning time scales when a user learns to operate a static vs. an adaptive interface. We show that this novel framework can be directly exploited to readily simulate a variety of interaction scenarios, to facilitate the exploration of the parameters that lead to optimal learning dynamics of the joint system, and to provide an empirical proof for the superiority of human-machine co-adaptation over user adaptation.

## Introduction

Interfaces between human and a machine are at the forefront of research in human augmentation [e.g., supernumerary limbs (Prattichizzo et al., 2014; Parietti and Asada, 2016; Yamen Saraiji et al., 2018), myoelectric prostheses (Antuvan et al., 2014; Wright et al., 2016; Dyson et al., 2018)], assistance [e.g., brain-computer interfaces (Santhanam et al., 2006; Millán et al., 2010; Nicolas-Alonso and Gomez-Gil, 2012; Jarosiewicz et al., 2015), brain-machine interfaces (Collinger et al., 2013), body-machine interfaces (Antuvan et al., 2014; Farshchiansadegh et al., 2014; Chau et al., 2017; Fall et al., 2017; Aspelund et al., 2020; Rizzoglio et al., 2020)], and rehabilitation (Rohm et al., 2013; Pierella et al., 2014; Donati et al., 2016).

In the majority of these applications, human-machine interfaces (HMIs) are expected to provide support to their users for prolonged periods of time. However, extensive usage requires interface stability, which is at present a considerable challenge both due to technological characteristics of the device, and due to physiological and functional processes active at the user's level (Young et al., 2011; Barrese et al., 2013; Orsborn et al., 2014; Downey et al., 2018). Co-adaptive algorithms for HMIs have been developed to address the issue of decoder instability (Vidaurre et al., 2011; Kao et al., 2017; Yeung et al., 2019; Degenhart et al., 2020; Silversmith et al., 2020) and to compensate for performance degradation due to the emergent closed-loop dynamics during use (Orsborn et al., 2012; Dangi et al., 2013; Shenoy and Carmena, 2014; Hahne et al., 2015; De Santis et al., 2018). One goal of these strategies is to reduce reliance on user adaptation to compensate for imperfections in the interface, a process that can be lengthy and cognitively demanding, besides being often insufficient for guaranteeing efficient control (Sadtler et al., 2014; Golub et al., 2018).

Despite the growing body of research, the majority of the efforts have been devoted to improving the decoding power of the algorithms while still little work has addressed the mechanisms that enable the user to learn an efficient control strategy to interact with the interface (Héliot et al., 2010; Kübler et al., 2014; Couraud et al., 2018; Perdikis and Millán, 2020). A few studies proposed to investigate user-interface co-adaptation through mathematical models in a two-learners setting as a viable way to study the system's learning trajectory. These models share the assumptions that (i) the user intention is known, (ii) the task goal is defined and accessible, (iii) the user and the interface act as independent agents that work together either to minimize some joint cost function (Müller et al., 2017), to minimize closed-loop error through joint stochastic optimization (Merel et al., 2013), to minimize an individual cost function in a game-theoretic formulation (Madduri et al., 2020), or to maximize the expected reward via reinforcement learning (DiGiovanna et al., 2009). These models are particularly suited for guiding and interpreting co-adaptation in the context of brain-machine interfaces. However, the requirements of knowing user intentions and task goals limits their application to situations when explicit information regarding the task objectives might not be directly accessible or user intentions cannot be reliably estimated. Moreover, there is no knowledge whether these models can be generalized to a task different from the one they have been trained on.

In order to tackle these limitations, here we propose a novel framework for studying co-adaptation in a human-machine interface setting when explicit information regarding the tasks goal might not be directly accessible. We draw inspiration from the field of body-machine interfaces, which have traditionally adopted what we may call general purpose-decoders and aim to identify a suitable low-dimensional encoding of the user's body signals to use for control in a variety of tasks. We propose a framework where the user and the interface are non-independent agents that co-adapt toward maximizing the interaction efficiency rather than optimizing task performance. We believe our approach is novel also in that it addresses the problem of non-stationarity in the user behavior together with learning through data that is not independently identically distributed, as it is generally the case in practical applications (Perdikis and Millán, 2020).

The framework defines a mathematical model of a user learning and a model of an adaptive body-machine interface. User learning is implemented through a strategy based on reward-weighted use-dependent learning (Diedrichsen et al., 2010) with the goal of generating actions that maximize the coherence with the associated sensory feedback over time. We use experimental data obtained from a previous study where participants interacted with a body-machine interface (De Santis and Mussa-Ivaldi, 2020) to validate the plausibility of the model. The interface, on the other hand, is modeled as a linear compression map from high-dimensional actions to low dimensional feedback, that adapts to minimize transmission loss through an unsupervised learning procedure (De Santis et al., 2018). We simulate the models in different scenarios to study the final performance and convergence of the system as a function of the learning time scales of both the user and the interface.

In the following sections, we provide a mathematical formulation for framing the problem of co-adaptation in the context of body-machine interfaces. In the first section, we provide details of the mathematical models for a generic interface user, a model for an adaptive interface, and of their interaction. We then describe the simulation scenarios developed to test the plausibility of the proposed model for the user and to evaluate the effect of the learning time scales of the user and the interface on the ability of the system to converge to a joint solution.

We provide a thorough interpretation of the results to show that this novel framework can be directly exploited (i) to readily simulate a variety of interaction scenarios, (ii) to facilitate the exploration of the parameters that lead to optimal learning dynamics of the joint system, and (iii) to provide an empirical proof for the superiority of human-machine co-adaptation over user adaptation.

## Problem Formulation

The control problem in a human-machine interface (HMI) scenario can be formulated as follows. The user has to control some physical or virtual device in order to perform a certain task. The interface implements a continuous map *B*, between a certain n-dimensional vector of inputs ***q*** generated by the user, and an m-dimensional output vector of controls to the machine, ***p***.

(1) $$p=B(q),\;B:{\mathbb{R}}^{n}\to {\mathbb{R}}^{m}$$

For instance, we may consider a user wanting to bring a computer cursor to a certain location on a screen. In this scenario, an interface may implement a map from body postures to the {x,y} location of the cursor on a screen (Mosier et al., 2005). Equivalently, another interface may define a transformation between the activity of neurons in the motor cortex and the velocity of the cursor (Santhanam et al., 2006).

As is often the case for HMI applications, we will assume that the dimensionality of the space of input signals recorded from the user is greater than the dimensionality of the signals necessary to control the device, *m* < *n*. This implies that not all inputs that the user generates will be equally effective in driving the device, as only vectors lying in the potent space of the map will determine a change in the output. Hence, when learning to operate the interface, a user is faced with both an explicit goal—to satisfy specific task requirements—and an implicit objective—to generate control signals that produce a change in the state of the device. We will call the space of all possible low dimensional vectors {***p***} different from the zero vector the *latent space* of the map.

For the sake of clarity, we will develop our formulation with application to body-machine interfaces, where *B* implements *a linear map and*, in particular*, an orthogonal transformation* between body postures and the state of the device (Farshchiansadegh et al., 2014). These particular properties allow us to derive a simplified and tractable mathematical formulation for the problem and to highlight interesting properties of the human-machine system that can extend beyond our particular case.

Considering the subset of linear orthogonal maps, for any given input vector ***q*** the corresponding set of output vector ***p*** can be defined as the orthogonal projection of ***q*** onto ℝ^*m*^:

(2) $$\begin{array}{r} p=Bq+B^{⊥}q \end{array}$$

Where *B*^⊥^ denotes the orthogonal complement of *B*, that maps the vector ***q*** into the zero vector or null space of *B*. Here *B* effectively defines an *m*-dimensional hyperplane embedded in ℝ^*n*^. Because the dimension of the null space is *n*−*m* > 0, the problem of identifying the inverse transformation of *B* is ill posed. Thus, we may expect the user to learn one out of the possible infinite particular solutions to the forward-inverse problem (Pierella et al., 2019).

Note that out of the possible generalized right-inverses of *B*, the pseudoinverse, also known as the Moore-Penrose inverse, *B*^†^ = *B*^*T*^(*BB*^*T*^)−1 represents the minimum norm solution in a least-square sense. In particular, for matrices with orthonormal columns, *B*^†^ = *B*^*T*^.

**Figure 1:**
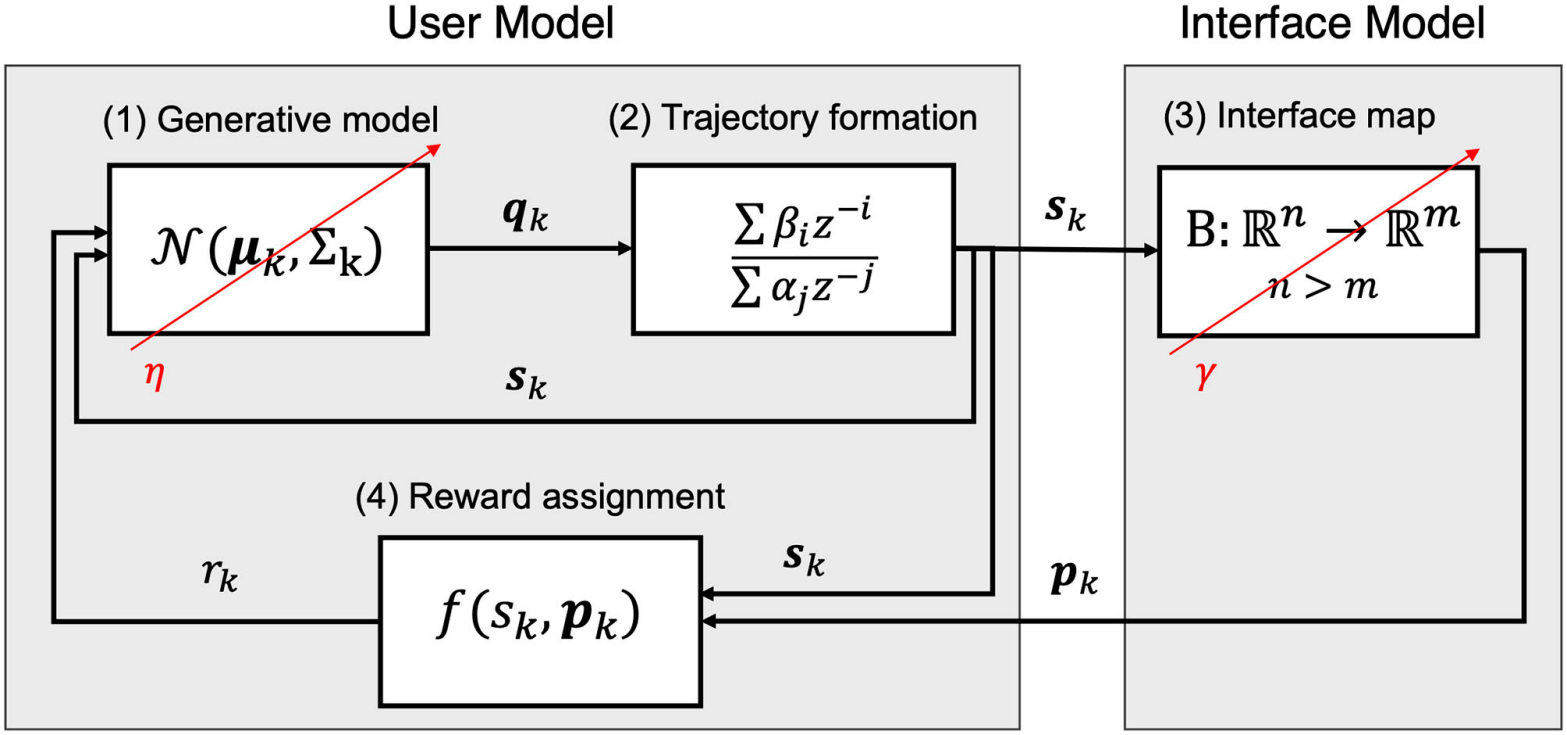
Model components and behavior. The user is composed of a generative model (1) that describes the probability of drawing a certain n-dimensional action ***q***_*k*_ at the instant *k*. The action ***q***_*k*_ contributes to generating a smooth trajectory of control signals ***s***_*k*_ for the interface (2). The interface map computes a vector ***p***_*k*_ of a dimension *m* < *n* and provides it to the user as feedback related to the smooth control action ***s***_*k*_ (3). The user model processes the feedback assigning a reward for the action based on the feedback (4). After each iteration, the map recursively updates its parameters based on the distribution of the observed user commands ***s***_*k*_ with learning rate γ. The user updates its generative model with learning rate η after a feedback is received, reinforcing the generated smooth action according to its reward.

Figure 1 summarizes the individual components of the proposed framework that will be described in the following sections. Section A Model for the User details the proposed model for a body-machine interface user that learns to interact with a static interface through a strategy based on reinforcement and use-dependent learning. Section A Model for an Adaptive Interface summarizes the proposed algorithm for implementing an adaptive body-machine interface. Finally, section A Model for User-Interface Coadaptation describes the algorithm for implementing user-interface co-adaptation within the proposed framework.

### A Model for the User

Let us assimilate a generic user to a generative process characterized by a probability distribution, $P_{u},\;$over the inputs ***q***. We will assume that the user is initially naïve to the interface, and that, with practice, will learn to control the interface up to a certain degree of proficiency. We then assume that learning will be reflected into a change in time of the probability of generating certain inputs based on the feedback received by the device (e.g., visual feedback of the cursor position).

Let us consider the case of $P_{u}$ following a multivariate Normal distribution with mean **μ∈**ℝ^*n*^ and covariance matrix Σ ∈ ℝ^*n* × *n*^. To account for time-dependency, we then assume that the distribution is non-stationary and can be summarized by a mean **μ**_**k**_ and a covariance matrix Σ_*k*_ at a certain discrete time *k*:

(3) $$P_{u}\left(k\right)\;\sim \;N\left(\mu_{k},\Sigma_{k}\right)\%(3)$$

In order to simulate how the probability distribution of the user's data changes with practice, we need to make certain assumptions as to what learning strategy the user might adopt. Previous work assumed the user follows an optimal control policy for directly minimizing task-related error (Merel et al., 2013, 2015; Müller et al., 2017). The authors of these studies rely on the knowledge of user intent for computing an error metric that guides an optimization routine over the model's parameters. However, the availability of the error depends on the capability to generate adequate input signals. Hence, when interacting with a system whose properties are still unknown, exploration of the input space is required (Bernardi et al., 2015; van Vugt and Ostry, 2019). Consistently, here we hypothesize learning in the early stages of interaction with the interface can be better approximated by a mechanism that acts through reinforcement and a memory of past inputs and their observed consequences. As a definition of error becomes unnecessary, the proposed approach allows framing the learning problem in a way that is task independent.

Let us assume that the user associates to every generated “action” ***q***_***k***_ a certain reward *r*_*k*_ based on the feedback received from the map and that the objective of the user is to learn to generate actions that maximize the expected reward over time:

(4) $$\{q_{k}\}:max_{\sum ,\mu ,B}\left\{E\left[r_{k}\right]\right\}$$

In particular, we assume that the reward assigned to each action is proportional to the “amount of feedback” the user receives for that action. For instance, actions that lie in the null space of *B* will receive zero reward, as they will produce no change in the state of the device, while actions that produce an observable change in state will receive a reward discounted by the amount of their null space component. The reward assignment rule, given (1), can be formalized as:

(5) $$r_{k}=\frac{tr\left(p_{k}p_{k}^{T}\right)}{tr\left(q_{k}q_{k}^{T}\right)}=\frac{tr\left(Bq_{k}q_{k}^{T}B^{T}\right)}{tr\left(q_{k}q_{k}^{T}\right)}$$

Note that the reward is a non-negative scalar, 0 ≤ *r*_*k*_ ≤ 1:

(6) $$\begin{array}{r} tr\left(Bq_{k}q_{k}^{T}B^{T}\right)=tr\left(q_{k}q_{k}^{T}\right),if||Bq_{k}||=||q_{k}\;||\\ tr\left(Bq_{k}q_{k}^{T}B^{T}\right)<tr\left(q_{k}q_{k}^{T}\right),if||Bq_{k}||<||q_{k}|| \end{array}$$

Equation (5) defines the reward as the amount of power transferred through the map and Equation (6) gives us the intuition that the reward is maximized if **q**_*k*_ lies in the potent space of *B* at every instant of time.

Let us now consider a set of samples {$\bar{q}$} generated over a finite time horizon [*k*_0_, *k*_1_] within which the generative model $p_{u}\left(k_{0}\leq k\leq k_{1}\right)\sim$ $N\left(\bar{\mu },\bar{\Sigma }\right)$ can be considered approximately stationary. Given (5) and knowing that $\bar{\Sigma }$ = $E\left[\left(\bar{q}-\bar{\mu }\right)\;{\left(\bar{q}-\;\bar{\mu }\right)}^{t}\right]=E\left[\bar{q}{\bar{q}}^{T}\right]-\;\bar{\mu }{\bar{\mu }}^{T}$, we can formulate the maximization policy for the expected reward in Equation (4) as follows:

(7) $$\begin{array}{l} E\left[r_{k}\right]=\frac{tr\left(E\left[\bar{p}{\bar{p}}^{T}\right]\right)}{tr\left(E\left[\bar{q}{\bar{q}}^{T}\right]\right)}\\ \;=\frac{tr\left(BE\left[\bar{q}{\bar{q}}^{T}\right]B^{T}\right)}{tr\left(E\left[\bar{q}{\bar{q}}^{T}\right]\right)}\\ \;=\frac{tr\left(B{\bar{\Sigma }B}^{T}\right)+tr\left(B\bar{\mu }{\bar{\mu }}^{T}B^{T}\right)}{tr\left(\bar{\Sigma }\right)+tr\left(\bar{\mu }{\bar{\mu }}^{T}\right)} \end{array}$$

We can find a more interesting expression for Equation (7) considering the set of input vectors centered in the mean: $\left\{q\right\}=\{\bar{q}\}-\bar{\mu }$. Knowing that $\bar{\Sigma }$ is symmetric and positive definite, we can define two matrices, a diagonal matrix Λ = *diag*([λ_1_, …, λ_*n*_]), where λ_*n*_ are the eigenvalues of $\bar{\Sigma }$, and an orthogonal matrix *V* = [**v**_1_|…| **v**_*n*_] with the corresponding eigenvectors as columns such that:

(8) $$\begin{array}{r} \bar{\Sigma }=V\Lambda V^{T} \end{array}$$

Using Equation (8), we can then rewrite Equation (7) in the case of random variables with zero mean:

(9) $$\begin{array}{r} E\left[r_{k}\right]=\frac{tr\left(B{\bar{\Sigma }B}^{T}\right)}{tr\left(\bar{\Sigma }\right)}=\frac{tr\left(BV\Lambda V^{T}B^{T}\right)}{\sum \lambda_{i}}=\frac{tr\left(C\Lambda C^{T}\right)}{\sum \lambda_{i}} \end{array}$$

where *C* = *BV*. We can immediately see from Equation (9) that the expected reward will be maximized if *CC*^*T*^ = *I*, hence if *V* = *B*^†^. This is equivalent to say that the reward will be maximized if the user learns to generate inputs that lie in the potent space of the interface map.

We have assumed the user could be modeled as a non-stationary generative process characterized by a certain expected value and covariance matrix at a certain instant of time. In the following paragraph we will now provide a mathematical formulation for iteratively computing the parameters of the user distribution based on the feedback received by the interface and the reward.

Given that the user receives feedback as a continuous stream, the distribution parameters should be estimated following an incremental approach. In the following, we will report the formulation originally proposed by Weng and colleagues (Zhang and Weng, 2001; Weng et al., 2003) for iteratively estimating the eigenvectors and eigenvalues of the data covariance matrix, that we have modified to account for non-stationarity in the distribution that generated the data (De Santis et al., 2018; De Santis and Mussa-Ivaldi, 2020). Every time a new sample ***q***_***k***_ is received, the sample estimates for the mean and the principal components of the covariance matrix can be updated as follows:

(10) $$\mu_{k+1}=\left(1-\eta \right)\mu k+\eta q_{k}$$

(11) $$w_{k+1}=\left(1-\eta \right)w_{k}+\eta \frac{\left(q_{k}-\;\mu_{k+1})(q_{k}^{T}-\;\mu_{k+1}\right)}{\left\|\;w_{k}\right\|}w_{k}$$

where **w**_*k*_**=**λ_*k*_**v**_*k*_ is the estimate of the eigenvector scaled by its corresponding estimated variance.

Equations (10) and (11) effectively implement a first order exponential smoothing filter with time constant τ = −*T*/ln(1−η), where *T* is the sampling interval and η the *learning rate*. Hence, the η parameter describes how fast new data is incorporated in the model or, equivalently, how quickly the user is willing to discount older memories. It has been suggested that the learning rate should be chosen within the range [10^−5^, 10^−1^] to ensure convergence and stability of the solution (Schmitt et al., 2016). By modulating the learning rate η we can characterize processes with a variable amount of memory and sensitivity to data that lie outside the distribution. In particular, small values of η will decrease the likelihood that new data will considerably affect the distribution parameters. This may be desirable when the reward for the current action is low. Conversely, the user should reinforce actions that are highly rewarded. This can be accounted for in the model by modulating the learning rate in proportion to the reward, as suggested by Diedrichsen et al. (2010):

(12) $$\begin{array}{r} \eta_{k}=\eta \cdot r_{k} \end{array}$$

According to Equation (12), at each iteration the learning rate will always be bounded between zero, whenever the current action receives zero reward, and η. It is interesting to note that for η_*k*_ = η the model would effectively mimic a process that is referred to as “use-dependent” or “experience-dependent” learning (Butefisch et al., 2000; Diedrichsen et al., 2010; Huang et al., 2011), which describes the progressive consolidation of patterns of activity by repeated occurrence of a same action.

The last component of the model that has to be addressed is how the process of action selection is carried out. In a real scenario, the user would select actions directed toward a goal, for instance to reach a target position with the computer cursor or when carrying out a pursuit task. As we are not interested in modeling the behavior of the user under specific task conditions, we will only include in the model the general requirement that the samples drawn by the user ought to be statistically dependent.

In practice, we simulate data to be dependent within a certain window *L* by filtering successive randomly drawn inputs {***q*_*k*_, …, *q*_*k*+*L*_}** with a first order autoregressive exponentially weighted moving average (ARMA) model, initialized with ***s***_0_**=*q***_0_:

(13) $$\begin{array}{r} s_{k}=\alpha s_{k-1}+\beta q_{k} \end{array}$$

**Figure 2:**
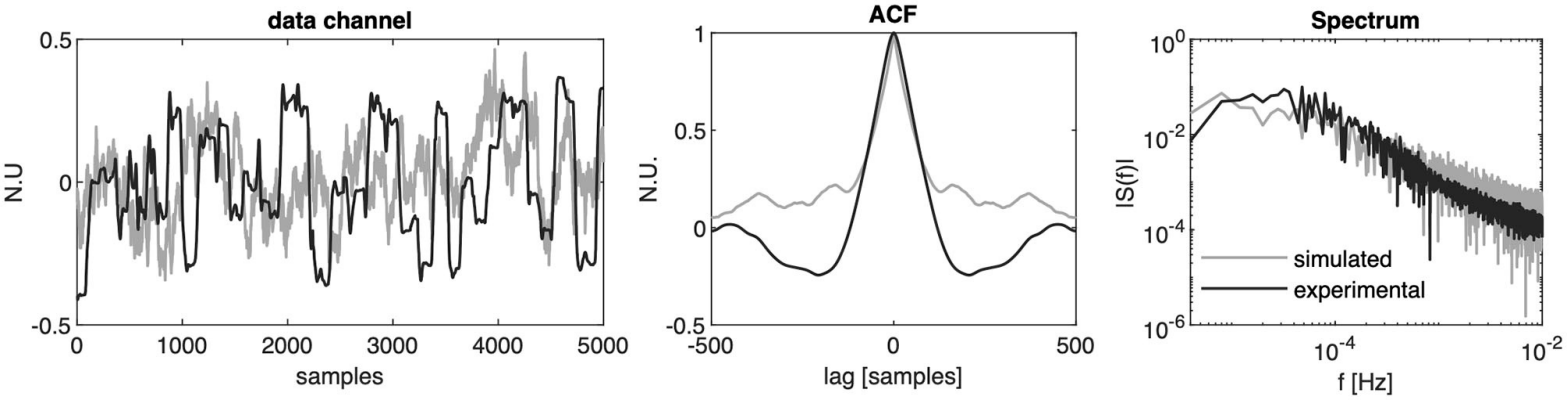
Experimental vs. simulated data. From left to right: normalized orientation data from one channel of IMU recordings while the user controlled the position of a 2D cursor in a reaching task with a body-machine interface (black line) and simulated sensor data channel extracted from a distribution with the same mean and covariance matrix (gray line); autocorrelation function of the experimental and simulated signal cropped at a lag of 500 samples; single-sided amplitude spectrum of the experimental and simulated signal assuming a sample frequency of 50 Hz.

The parameters values α = 0.99, β = 0.15 have been chosen to resemble the correlation encountered in experimental data sequences from upper-limb movements after centering the data in the mean (De Santis and Mussa-Ivaldi, 2020). An example of real vs. simulated data is shown in Figure 2.

Finally, we have to add a regularizing term that encodes some constraints on the structure of the variance of the generated action that, under conventional circumstances, would be induced by task requirements. Given that the model generates correlated data, the possibility for the simulated user to successfully learn to generate actions that increase the expected reward depends on the specific sequence of actions the user produces. In fact, it is likely that the model in the present form will learn degenerate solutions. For example, the user may learn to consistently produce actions along a line parallel to one column of the map rather than in two dimensions. Another, less intuitive, singularity relates to the magnitude of the actions the user learns. As we assume that samples generated by the user are normally distributed, actions that lie closer to the mean are more likely to be produced than actions that lie further from the mean. Consequently, we can predict from the recursiveness of Equations (10) and (11) that the user distribution will progressively shrink until the variance becomes zero.

In order to prevent the user from learning degenerate solutions and to avoid the problem of the “vanishing variance,” we need to introduce two regularizing constraints on the structure of the covariance matrix used to generate data at each step. In particular, we assume here that the user is motivated in producing actions that span (at least) two dimensions. This motivation is then translated into a corrective term for the variance matrix Λ_*k*_ at each time step:

(14) $$\begin{array}{r} {\hat{\Lambda }}_{k}=diag\left(\left|\lambda_{k}+\lambda_{C}+z\right|\right) \end{array}$$

where **λ**_*C*_ is a n-dimensional vector of corrective factors and **z** is a n-dimensional vector of random noise.

The first constraint, formalized in Equation (15), imposes that the vigor of the user's actions in the task dimensions over time has to remain constant. No constraint is imposed on the cumulative variance distributed along the remaining dimensions.

(15) $$tr\left(\sum_{j=1}^{m}\lambda_{j,k}\right)=tr\left(\sum_{j=1}^{m}\lambda_{j,0}\right)$$

The second constraint imposes that the percentage of variance accounted for by the first *m* eigenvalues does not decrease compared to the initial condition.

(16) $$\begin{array}{r} \frac{\Lambda_{k}}{tr\left(\Lambda_{k}\right)}\geq \frac{\Lambda_{0}}{tr\left(\Lambda_{0}\right)} \end{array}$$

### A Model for an Adaptive Interface

In order to simulate interface adaptation, we refer to the algorithm initially proposed in De Santis et al. (2018). The goal of interface adaptation is to incrementally adjust the interface map to better resemble the distribution of the user's action while the user is controlling the interface. If we recall Equation (9), the optimal solution to the control problem in terms of reward maximization is given by *BV*(*BV*)^*T*^ = *I*. If the interface is static, this can be achieved only if the user learns to generate data points that lie in the potent space of the map. However, this process is likely to develop over a prolonged period of time as a result of the interaction of multiple learning mechanisms with task constraints (Rohde et al., 2019) and even then, the user may learn a solution far from the one with minimum norm.

A simple way to speed up the process is to steer the interface toward finding a better low-dimensional approximation of the user's input covariance. We can then reformulate Equation (11) to iteratively update the orthogonal components of the map to resemble the first eigenvectors of Σ_*k*_:

(17) $$\begin{array}{r} b_{k+1}=\left(1-\gamma \right)b_{k}+\gamma \frac{q_{k}q_{k}^{T}}{\left||b_{k}|\right|}b_{k} \end{array}$$

where ***b*** is the first column vector of *B*. The expression can be easily generalized to multiple orthogonal components but, for brevity, we let the reader refer to Weng et al. (2003) for a detailed formulation. Equation (17) in its form assumes that ***q***_***k***_ comes from a distribution with zero mean. In the following, we will assume without loss of generality that this condition is true.

### A Model for User-Interface Coadaptation

In this section, we provide a mathematical modeling of a user coupled with an adaptive interface. In the context of the proposed framework, both the user and the interface aim at maximizing the transfer between a user-generated input ***q***_*k*_ and its low dimensional counterpart ***p*_*k*_**. However, the user follows a strategy driven by reward maximization, while the map simply tries to approximate the covariance of the user's generative process. The algorithm for implementing user-interface coadaptation is summarized in Table 1.

**Table 1:** Coadaptation.

Define: η, γ, *K*	
Initialize model parameters	$\Sigma_{0}=\bar{\Sigma },$ μ_0_ = **0**, *B*_0_=[***b***_1, 0_|***b***_2, 0_];
**for** *k* in [1, *K*], **do:**	
Draw an independent input:	$q_{k}\sim N\left(0,\Sigma_{k}\right)$
Update the sample trajectory (Equation 13):	***s***_*k*_ = α***s***_*k*_+β***q***_*k*_
Project the data onto the map (Equation 1):	_*p*_*k*_ = *Bk*_*s*_*k*_
Assign the reward (Equation 5):	$r_{k}=\frac{tr\left(p_{k}p_{k}^{T}\right)}{tr\left(s_{k}s_{k}\right)}$
Update user parameters (Equation 11):	*w*_*k*+1_←(1−*ηr*_*k*_)***w***_*k*_+*ηr*_*k*_*f*(*s*_*k*_, ***w***_*k*_)
Compute eigenvectors and eigenvalues:	***v***_*k*+1_ = ***w***_*k*+1_/||***w***_*k*+1_||; λ_*k*+1_ = ||***w***_*k*+1_||;
Compute regularized variance (Equation 14):	${\hat{\Lambda }}_{k+1}=diag\left(\left|\lambda_{k}+\lambda_{C}+z\right|\right)$
Update the generative model (Equation 8):	$\Sigma_{k+1}=V_{k+1}{\hat{\Lambda }}_{k+1}V_{k+1}$
Update map parameters (Equation 17):	***b***_*k*+1_←(1−γ)***b***_*k*_+*γf*(***s***_*k*_, ***b***_*k*_)
**end for**	

The ability of the joint system to converge to a solution largely depends on the choice of the learning rate parameters for the user and the map, η and γ. Previous theoretical work suggested that imbalanced learning rates are more likely to converge to a stable equilibrium (Igual et al., 2019) and that interface learning rates that are too high quickly lead the joint system to instability and prevent the user from adapting (Hahne et al., 2015; Müller et al., 2017). Tuning the learning rate of the interface to find the ideal trade-off between speed and stability is often impractical especially when the learning rate of the user is unknown. The simulations proposed in the next sections aim to contribute some theoretical guidance for implementing interface adaptation and the consequences of parameters choices on the joint system convergence that find direct application to body-machine interfaces.

## Simulation Scenarios

In this section we analyze two scenarios in order of complexity. We first examine the simulated user behavior in relation to the choice of the learning parameter when interacting with a stationary interface. We also verify that our model is sufficient to explain experimental data. We then propose simulations to characterize the behavior of the joint user-interface system for a range of possible learning parameters.

### Simulating a User Learning a Stationary Interface

As previously mentioned, we assume that the user can be assimilated to a generative process with zero mean and a non-stationary covariance matrix Σ that generates a sequence of dependent samples through the ARMA model in Equation (13). The dimensionality of the input data and the interface have been chosen to match the ones in our previous experimental study, where 10 individuals learned to control a 2D cursor moving their upper limbs (De Santis and Mussa-Ivaldi, 2020). In the study, participants first performed 60 s of random arm movements and then a reaching task with the interface. Each participant interacted with a customized interface, initialized to the first two eigenvectors extracted applying principal component analysis to the dataset of random motions.

Consequently, here the dimension of the user input was set to eight and the dimension of the feedback to two, leaving the simulated user with 6 redundant dimensions. The interface map *B* is 2 ×8 rectangular matrix and was chosen identical to the map the participant interacted with during the experiment. The user model covariance matrix was instead initialized using the first 60 s of sensor data recorded when the same subject first performed the reaching task.

We ran multiple simulations with 20 different values of learning rate logarithmically spaced between 10^−4^ and 10^−1^ over a 40k samples horizon, which roughly correspond to an experimental session of 10–15 min. Since the trajectory of the simulated user is dependent on the random sequence of samples that are generated, each simulation was repeated 20 times with different random seeds. The variance of the additive random noise term in Equation (14) was chosen to be 10^−4^.

The simulation steps are summarized in Table 2.

**Table 2:** User learning.

Define: *B, K* = 10*k*	
**for** n in [1,10], **do:**	
**for** η in *list*, **do:**	
Initialize model parameters	$\Sigma_{0}=\bar{\Sigma },$ μ_0_ = **0**, *B*_0_=[***b***_1, 0_|***b***_2, 0_];
**for** *k* in [1, *K*], **do:**	
Draw an independent input:	$q_{k}\sim N\left(0,\Sigma_{k}\right)$
Update the sample trajectory (Equation 13):	*s*_*k*_ = α*s*_*k*_+β*q*_*k*_
Project the data onto the map (Equation 1):	_*p*_*k*_ = *Bk*_*s*_*k*_
Assign the reward (Equation 5):	$r_{k}=\frac{tr\left(p_{k}p_{k}^{T}\right)}{tr\left(s_{k}s_{k}\right)}$
Update user parameters (Equation 11):	*w*_*k*+1_←(1−*ηr*_*k*_)*w*_*k*_+*ηr*_*k*_*f*(*s*_*k*_, *w*_*k*_)
**end for**	
**end for**	
**end for**	

We then asked whether the model was able to fit the actual user distribution parameters recorded during the reaching task. We modified Table 2 to take as input a sequence of 40k samples from the experimental data rather than asking the model to generate its own. As the assumption of the data having zero mean does not hold in this case, we simulated a non-centered user model. Since in this condition the reward associated to each sample is predefined, the model's behavior is deterministic. Accordingly, the model was simulated only once for each learning rate.

### Simulating User–Interface Coadaptation

In this scenario, both the user and the interface parameters are allowed to change following the steps described in Table 1. In order to simulate a more realistic condition, we implemented the user as a generative process that outputs statistically dependent data according to Equation (13).

We simulated all the possible combinations for the learning rate of both the user and the interface considering 20 log spaced values between 10^−4^ and 10^−1^. Hence, we trained a total of 400 models over 40,000 iterations 20 times, each using different random seeds.

The interface map and the virtual user's distribution parameters were once again initialized using participant #S8 as a reference, to allow comparing the results across the different scenarios.

### Performance Metrics

We computed three metrics to assess the evolution of the user covariance manifold in relation to the interface map across samples. These metrics are commonly used to assess user learning in body-machine interfaces as well as other redundant control tasks (Ranganathan et al., 2014; Thorp et al., 2017; De Santis et al., 2018).

1. Planarity: quantifies the amount of variance that the simulated user distributes in two dimensions. If the user is effectively learning to maximize the reward over time (that is amount of data that project onto the potent space of the map) we expect the user to progressively reduce the probability of generating actions along the dimensions associated with a null feedback. Experimental data confirmed that body-machine interface users learn to increase planarity in a 2D task (Ranganathan et al., 2013, 2014; De Santis and Mussa-Ivaldi, 2020). Planarity varies between 0 and 1 and is computed at each iteration from the variance of the user generative model as follows:
  $$\begin{array}{l} \frac{\lambda_{1,k}+\lambda_{2,k}}{tr\left(\Sigma_{k}\right)} \end{array}$$
2. Subspace Angle [deg]: is a measure of angular distance between subspaces and is used here to quantify how close the user distribution is to the interface map. Two maximally tangent (parallel) subspaces will have a Subspace Angle close to 0 deg, while 90 deg indicated that the two subspaces are orthogonal and share a minimal projection. It is computed as the angle between the hyperplane described by the map and the hyperplane described by the first two principal component of variance extracted from the user distribution at each iteration:
  $$\begin{array}{l} \cos^{-1}\left(\left|{\;B}_{k}^{T}\cdot \left[V_{1,k}|V_{2,k}\right]\;\right|\right)\cdot 180/\pi \end{array}$$
3. VAF: Variance Accounted For by the interface map, varies from 0 to 100% and quantifies the percentage of user covariance that is transferred to the feedback though the interface map. This metrics effectively encodes the average reward associated with the current user distribution and can be considered a measure of control efficiency. It is computed every iteration as:
  $$\begin{array}{l} \frac{tr\left(B_{k}\Sigma_{k}\right)}{tr\left(\Sigma_{k}\right)}\cdot 100 \end{array}$$
4. Rate of convergence [samples]: quantifies the number of iterations needed for the user to improve performance by 63% while converging toward a stable solution either independently or jointly with an adaptive interface. It is computed over the values of Subspace Angle between the user and the interface over training as the time constant of the single exponential function that yields the best least square fit to the data.

In order to evaluate the proposed metrics over the experimental data, the parameters of the covariance matrix through time were estimated using a sliding window of 3,000 samples (60 s of data) over the recorded data sequence.

## Results

### Simulating a User Learning a Stationary Interface

Here we consider the effect of the choice of the learning rate parameter on the ability of a simulated user to maximize the expected reward over time when interacting with a static map (γ = 0). We will first consider the plausibility of our simplified model of user learning by testing its ability to emulate the performance metrics extracted by the experimental data recorded from 10 actual users of our previous study (De Santis and Mussa-Ivaldi, 2020). Then we will analyze the results obtained after training an array of 20 user models with varying learning rates to interact with a static interface.

#### Emulating a Real User

The generative portion of the model has been replaced by a sequence of experimental data and we evaluated the ability of the model to fit the performance metrics computed from the users' data.

**Figure 3:**
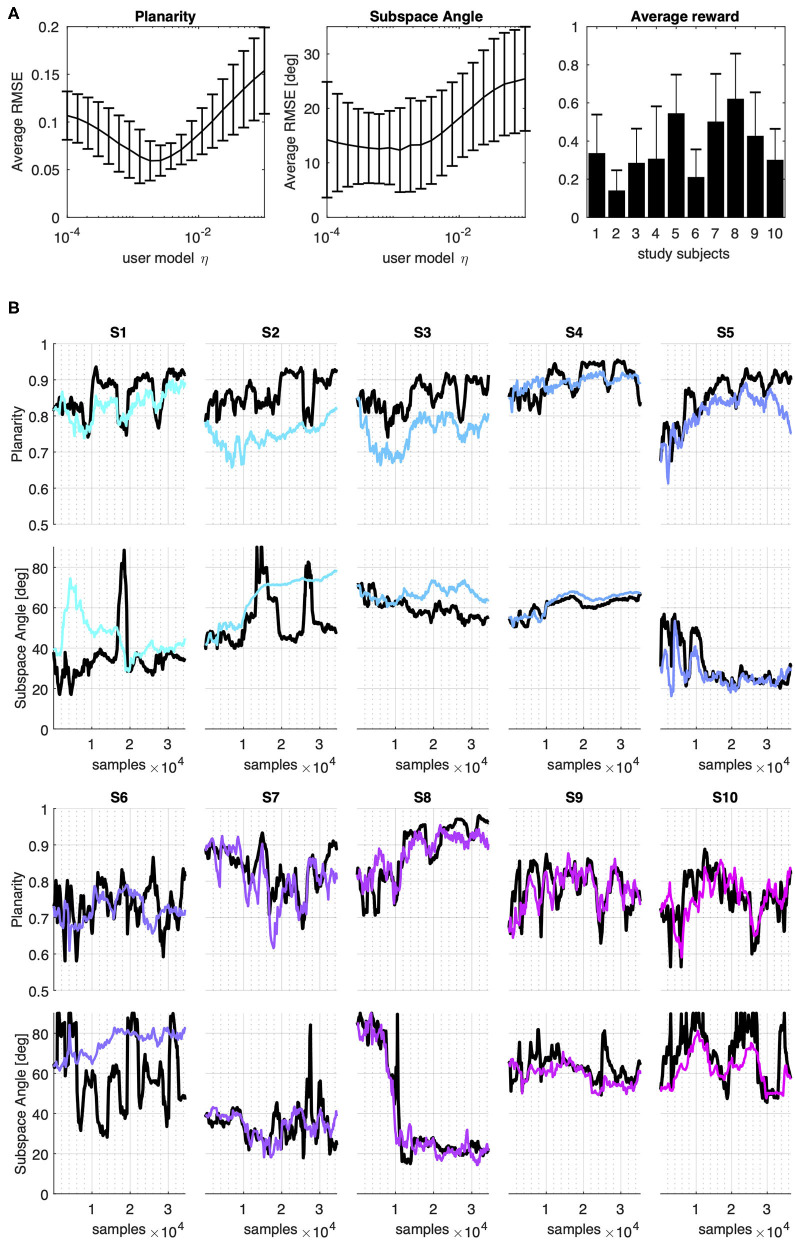
Model prediction of experimental data. Planarity and Subspace Angle estimated from the data of 10 study participants from De Santis and Mussa-Ivaldi (2020) **(A)**: from left to right, average performance of the model vs. experimental datasets in terms of Root Mean Squared Error (RMSE) on Planarity and Subspace Angle metrics and the average reward computed over the dataset given the user interface map. **(B)** Model performance across iterations. Metrics computed on the experimental data are reported in black solid lines. Colored lines represent the values obtained from the data distribution generated iteratively by one out of 20 models with varying learning rates that have been trained with experimental data. The selected models in the figure have learning rate of 0.0013.

Figure 3 summarizes the performance of the user model trained on data from 10 individuals controlling a body-machine interface with the upper limbs. Figure 3, panel A compares the average performance of the model over the 10 experimental datasets in terms of Root Mean Squared Error (RMSE) on Planarity and Subspace Angle metrics computed on simulated and experimental distributions and the average reward computed over the dataset given the interface map.

The model that achieved the minimum average RMSE on both metrics was found for η = 0.0013 which corresponded to an average error on Planarity of 0.0639 ± 0.028 and 12.35 ± 7.72 deg on the Subspace Angle (mean ± standard deviation).

The evolution of Planarity and Subspace Angle across iteration for all the 10 participants considered is depicted in Figure 3, Panel B, where the solid black lines represent the metrics computed from experimental data, while the colored line the metrics computed from the model's distribution with η = 0.0013. From the figure, we can see that the behavior across participants varies greatly, both in terms of the evolution of the distribution metrics and the reward computed a-posteriori on the data. Nevertheless, the model allows to closely follow the course of Planarity and Subspace Angle in time.

#### Simulating a Virtual User

**Figure 4:**
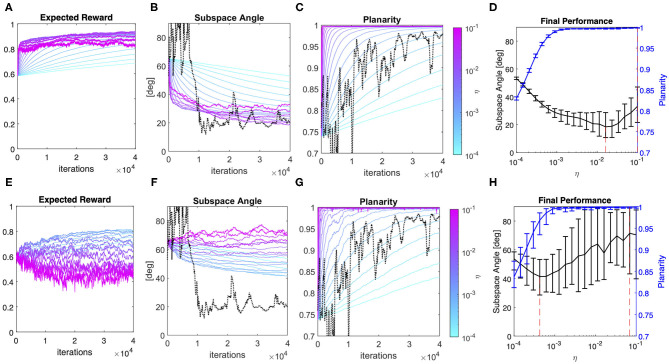
User Model simulation: independent vs dependent data. Average expected reward, planarity, and subspace angle over 20 simulation runs of a model with varying learning rates generating a sequence of 40k independent (top row) or dependent (bottom row) data. Each model was simulated to evolve from the same initial condition as participant S8. **(A,E)** Expected reward computed over the generated sequences of independent and dependent data, respectively and averaged over the 20 simulation runs; **(B,F)** Subspace Angle between the 2D approximation of the simulated user's covariance and the interface map at each learning iteration for independent and dependent samples, respectively; **(C,G)** Planarity index computed from the simulated user's covariance matrix at each learning iteration for independent and dependent samples, respectively. The range of the learning rate parameter spanned an interval from to and the relative performance of the model is identified by a color that changes from light blue to pink for increasing learning rates, as indicated by the color bar. The dotted lines overlay the experimental metrics obtained from #S8, also reported in Figure 3; **(D,H)** summary of Planarity (blue line) vs. Subspace Angle (black line) obtained in the final 200 learning iterations at each learning rate in the case of independent and dependent samples, respectively. The solid lines represent the average value computed over the 20 simulation runs for each model, while the dispersion indicates the 95% confidence interval. The red dotted lines mark the best performing model for the two parameters considered.

After having verified the plausibility of our model, we simulated a virtual user in a body-machine interface scenario using the algorithm outlined in Table 2 while varying the fixed component of the learning rate, the parameter η. We compared the impact of assuming that the user learns though a sequence of independent vs. dependent data inputs. Figure 4 summarizes the results of the simulations.

Panels A and D show how the expected reward changes across iterations as a function of the user distribution and the interface map. The results show that assuming data to be dependent does not affect the course of Planarity of the generative model covariance. However, this assumption greatly impacts the distance between the model distribution and the interface map. As suggested by the difference in the values of Subspace Angle obtained in the case of independent (Figure 4, panel B) vs. dependent observations (Figure 4, panel E), random exploration leads to solutions that lie closer to the subspace identified by the interface map.

A first important observation is that the assumption that the data is independently distributed affects the relationship between the learning rate parameter and model convergence. From Figure 4 (panels D and G) we notice that the range of values of the learning rate parameter that yield better final performance is reduced in the case of dependent data, with smaller learning rates leading to an overall better performance. In our simulations, the model that drew nearest to the subspace identified by the interface map was found for η = 0.0162 in the case of independent observations, and η = 0.0004 for dependent data sequences. Moreover, accounting for data dependency determined more than a 3-fold increase (3.2 ± 1.7) in the variability of the final solution across simulation runs (Figure 4, panels D vs. G).

This observation finds its counterpart in the experimental evidence that every user develops a unique solution to the interface control problem, as exemplified by Figure 3, Panel B. In fact, given a same model starting from the same initial condition, divergent results can be obtained for different data sequences. This can be seen comparing the results obtained for the two sequences of user's playback and simulated data in Figures 3, 4 and from Supplementary Video 1, that shows the complete evolution of VAF, Planarity, and Subspace Angle in each of the 20 simulation runs as the learning rate of the user increases from 10^−4^ to 10^−1^.

In summary, these results suggests that (i) the particular solution an interface user may converge to depends strictly on the patterns of input covariance generated during learning and that (ii) it is virtually impossible to accurately predict the learning trajectory of an interface user unless the exact sequence of control actions is known.

### Simulating User-Interface Coadaptation

**Figure 5:**
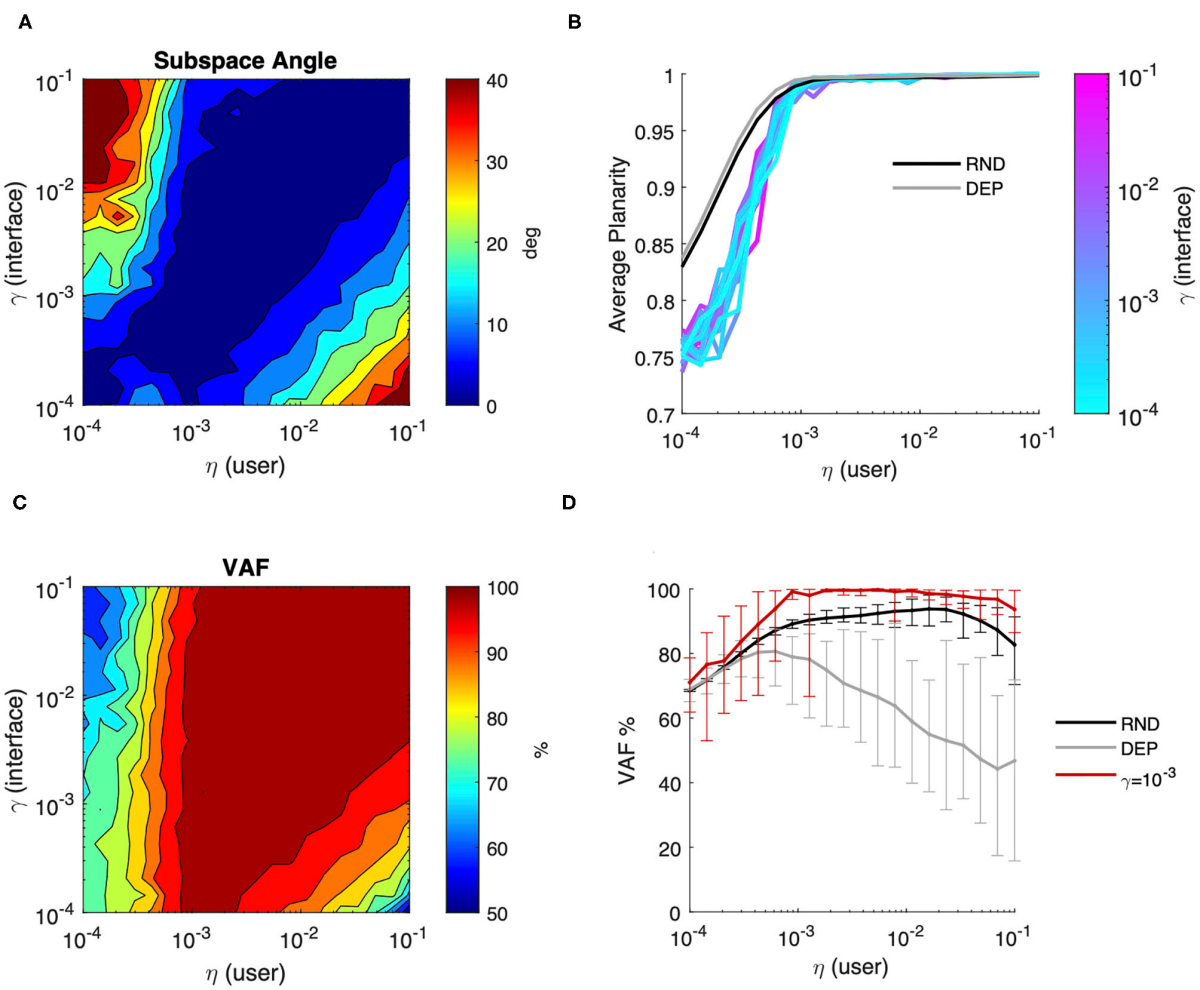
User-interface joint performance after co-adaptation. Final performance computed as the average value over the final 200 iterations of joint learning across all combinations of user (η) and interface (γ) rates of adaptation; **(A)** average Subspace Angle between the maximum variance 2D approximation of the user's covariance and the interface map. Levels indicate steps of 5 degrees; **(B)** average Planarity (colored lines). The x-axis represents the learning rate of the user, while the color represents the interface adaptation rate. The gray and black lines represent the final Planarity obtained in the case of user learning with dependent and independent samples, respectively; **(C)** average Variance Accounted For by the interface map. Levels indicate steps of 5%; **(D)** comparison of the average percentage of variance accounted for by the interface for different degrees of user learning in the case of a static interface and independent samples (RND—black line) or dependent samples (DEP—gray line) and after interface coadaptation with γ = 10^−3^ (red line). The error bars represent the 95% confidence interval over 20 simulation runs.

This section summarizes the results obtained simulating a set of user-interface dyads with different combinations of the respective learning rate parameters η and γ. Here we assumed that the user generates a sequence of dependent inputs, and both the user and the interface adapt their parameters every iteration step. The interface's goal is to minimize information loss and the user's implicit objective is to maximize the expected reward over time through action reinforcement. Results obtained during co-adaptation are contrasted with simulation results of user learning with dependent and independent data sequences but without interface adaptation in terms of (i) final performance (Figure 5) and (ii) the evolution of the solution over time (Figures 7, 8).

Figure 5 summarizes the final performance of the user model relative to the interface as a function of the learning rate of the two processes and provides a visual comparison between the performance achieved by user learning alone and by user-interface co-adaptation. As we can see from Figure 5—panel A, in the end of the simulation the user model and the adaptive interface learn to encode very similar subspaces for a broad combination of user/interface learning rates. In general, the best performance was achieved when the user and the interface adopted comparable learning rates (η_*i*_ = γ_*i*_: 0.87 ± 2.0 deg), while the least favorable conditions occurred when a fast-adapting interface (γ ≥ 10^−2^) was combined with a very slow learner (η < 10^−3^) and whenever a very fast learner was paired with a slow-adapting interface. Notably, co-adaptation yielded considerably smaller Subspace Angle on average (11.5 ± 12.6 deg) compared to a user learning through dependent data sequences for any combination of learning rates (≥40 deg on average, see Figure 4—panel G).

Contrarily to the distance between subspaces, Planarity of the user's generative model (Figure 5—panel B) was not dependent on the rate of model adaptation. Compared to the case of a user learning a static map, planarity during co-adaptation developed on average to a lesser extent only for very slow learning rates (η < 0.0005).

The global effect of co-adaptation on the control efficiency in terms of Variance Accounted for by the interface is summarized in Figure 5—panel C, while panel D compares the control efficiency during no map adaptation vs. γ = 10^−3^. The figures show that even a relatively small degree of interface co-adaptation significantly improves the VAF compared to user's solo learning. More in general, we observe that co-adaptation yields a variance accounted for by interface map of 90% on average with the exception of user-interface dyads composed of very slow learners. This latter point should not be surprising, given that slow learners paired with a sufficiently slow-adapting interface can learn to accurately approximate the subspace spanned by the interface map, but are unable to sufficiently minimize variance in non-relevant dimensions, as shown by low levels of distribution Planarity.

**Figure 6:**
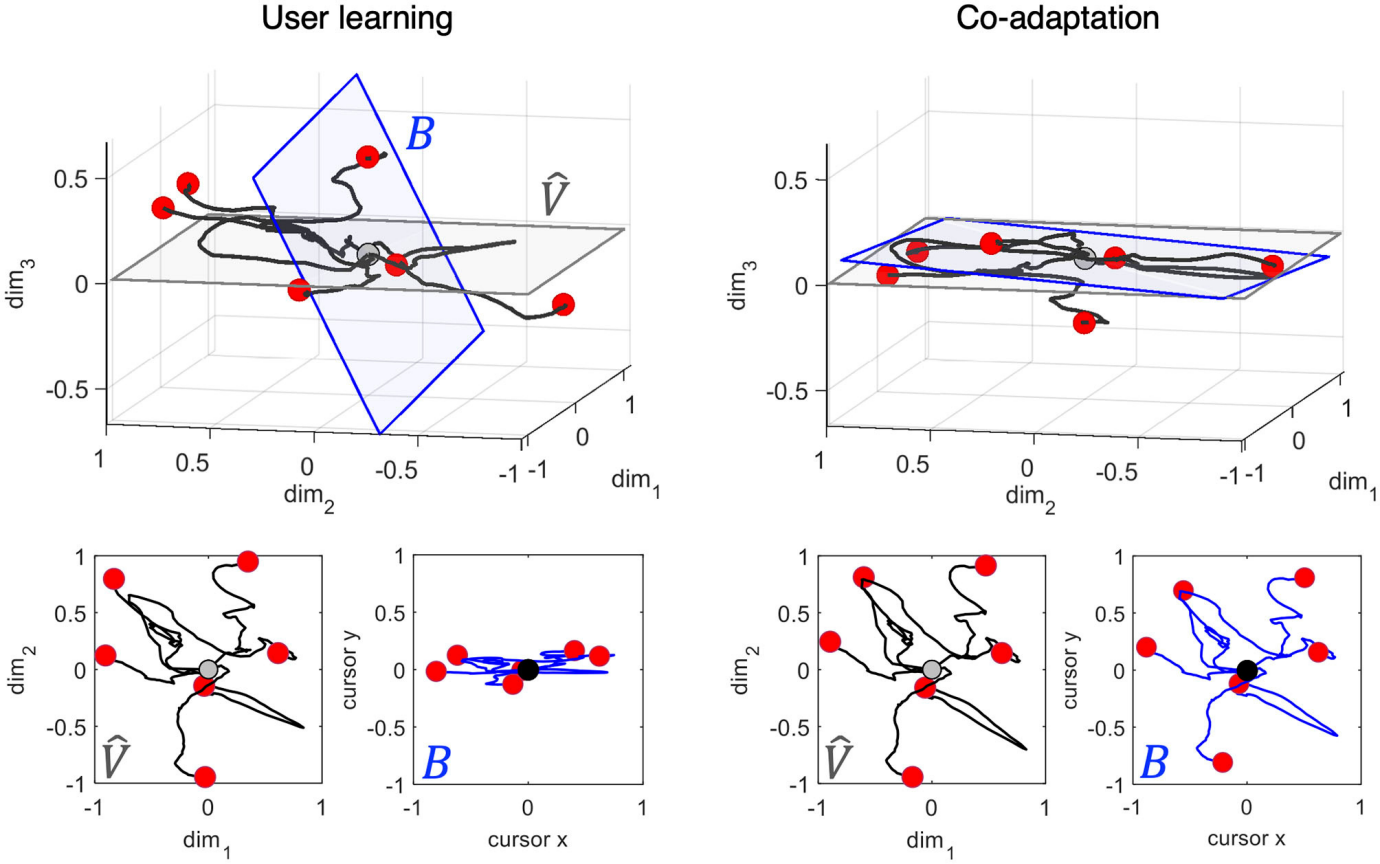
Control efficiency after user learning vs. co-adaptation. Projection of six simulated smooth trajectories in the 8D space of the sensors into (top panels) the first three eigenvectors of the user model movement distribution, (bottom panels) the first two eigenvectors of the user model movement distribution and the corresponding 2D cursor movement at iteration 1000. The user model was trained with η = 0.0013 in both conditions, while the interface had γ = 0 in the user learning condition, and γ = 0.0013 during co-adaptation. The trajectories in body space are depicted in black, while the corresponding cursor trajectories are depicted in blue. All trajectories start from the origin. Red circles denote the end of a trajectory (the equivalent of a target point). The blue hyperplane *B* represents the subspace of the cursor coordinates defined by the interface map, the gray hyperplane represents the subspace spanned by the first two components of body movement variance ($\hat{V}$). The subspace angle between *B* and $\hat{V}$ was 69.12 deg in the User Learning condition and 21.79 deg in the Co-learning condition at iteration 1000.

Figure 6 provides an intuition of the impact of user-interface co-adaptation on control efficiency when controlling a 2D cursor through the body-machine interface. We simulated a set of six smooth trajectories in the 8D space of the sensors, that we call body trajectories. These trajectories were then mapped into 2D cursor trajectories by the interface map B. For the sake of visualization, we limit ourselves to consider the 3D subspace spanned by the first three components the body movement at a certain iteration *k* [the projection of the body movement along (**v**_1, *k*_, **v**_2, *k*_, **v**_3, *k*_)]. This subspace is depicted in the top panels of Figure 6. The blue hyperplane *B* represents the subspace of the cursor coordinates defined by the interface map, the gray hyperplane represents the subspace spanned by the first two components of body movement variance ($\hat{V}$). The two planes correspond to *B* and $\hat{V}$ computed at *k* = 1000 from a model of a user learning with η = 0.0013 vs. a model of user-interface co-adaptation with η = γ = 0.0013. The six body trajectories are shown in black starting from the center (gray circle) and ending each in a red circle. The bottom panels show the projection of the body trajectories on $\hat{V}$ (in gray) and on *B* (in blue). As previously noted, user-interface co-adaptation allows maximizing the amount of movement that projects into cursor displacements compared to user learning, yielding to a minimal distortion between the intended and perceived trajectory. The subspace angle between *B* and $\hat{V}$ was 69.12 deg in the User Learning condition and 21.79 deg in the Co-learning condition at iteration 1000. The figure shows how coadaptation can be advantageous from the standpoint of interface controllability already early in the traning.

**Figure 7:**
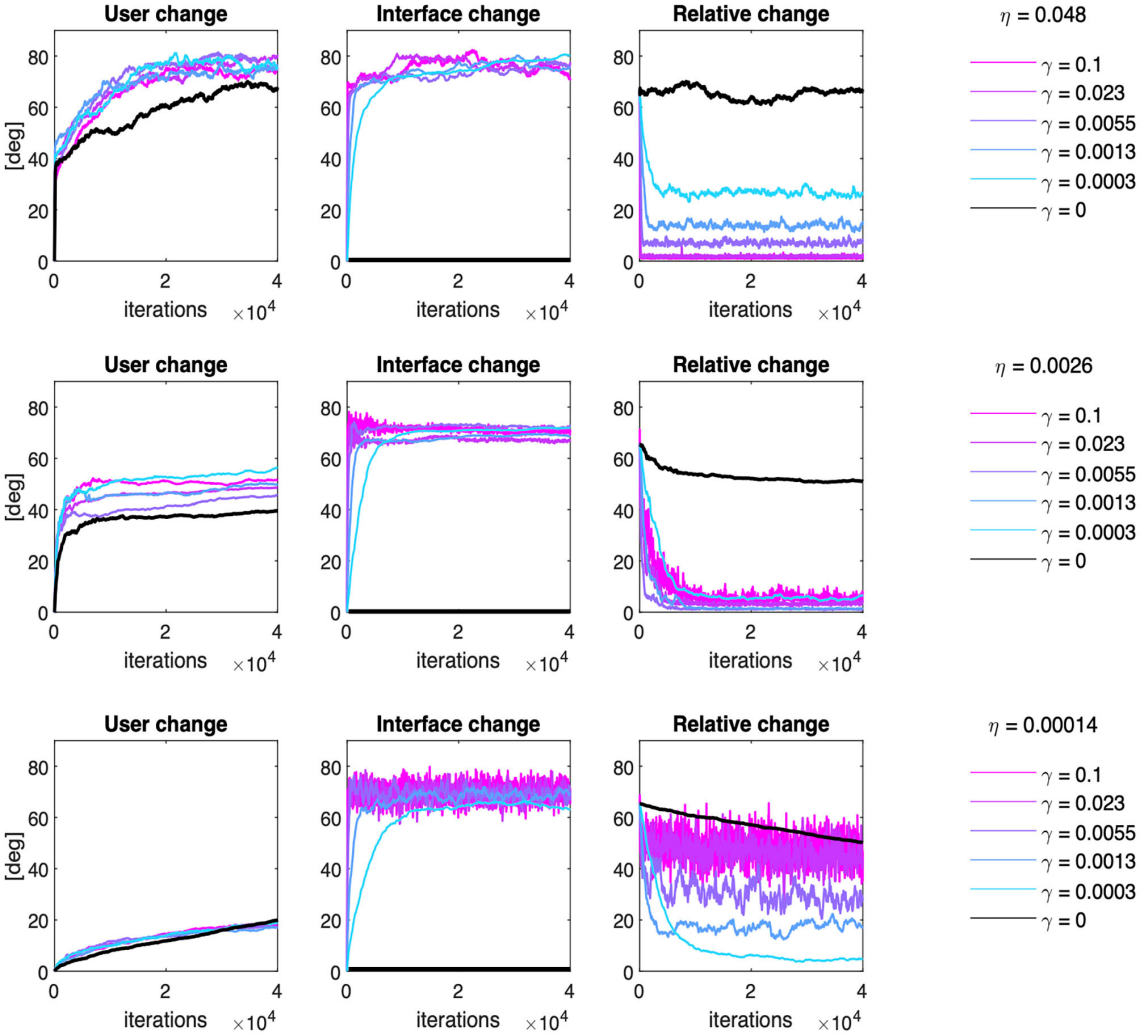
Co-adaptation: examples of user and interface evolution though time. Effect of six different interface adaptation rates on the Subspace Angle of the simulated users relative to the initial condition (leftmost column), of the interface relative to the initial condition (middle columns) and the relative angle between the interface and the user at each training iteration (rightmost column). The effect of co-adaptation has been exemplified here for a slow-learning user (η = 0.00014, bottom row), a user with intermediate learning rate (η = 0.0026, middle row), and a fast-learning user (η = 0.048, top row).

Figure 7 provides a series of examples regarding the effect of co-adaptation on the average time course of the Subspace Angle between the user and the interface over training iterations. Three main points should be highlighted.

Firstly, interface co-adaptation does not eliminate the need for user learning. In fact, Figure 7 (left column) shows that amount of change in the 2D subspace containing most variance of user input induced by learning is generally greater when the user interacts with an adaptive interface (colored lines), rather than a static interface (black line).

Secondly, user-interface co-adaptation yields better performance than user learning alone, as the relative distance tends to zero for suitable choices of interface adaptation rate (Figure 6, right column). In particular, adaptation rates that are too small tend to steer the system toward sub-optimal solutions (e.g., in the case of η = 0.048 and γ = 0.0003). Whereas adaptation rates that are too high induce not only suboptimal convergence, but also instability in the joint solution as the variability in the relative angular change grows proportionally with γ (e.g., in the case of η = 0.00014 and γ > 0.0003).

Lastly, the particular choice of γ seems to have a very marginal influence on the amount of change in the interface map compared to the initial condition (Figure 7, middle column). A two-way ANOVA considering the user and interfaces learning rates as independent factors over the 20 independent runs found that the amount of interface change is heavily dependent on the learning rate of the user [*F*_(19, 7600)_ = 44.29, p < < 0.001], with greater change induced by faster-learning users (about 10 degrees more than slower users). The effect of the interface change is more marginal [*F*_(19, 7600)_ = 2.14, p = 0.003] mostly due to the high variability across repetitions, as a *post-hoc* test with Bonferroni correction only identified one significant comparison at the significance level of 0.01 (γ = 0.0001 *vs*. γ = 0.0055). No effect of the interaction between user and interface learning rates was found [*F*_(361, 7600)_ = 1.05, p = 0.233].

**Figure 8:**
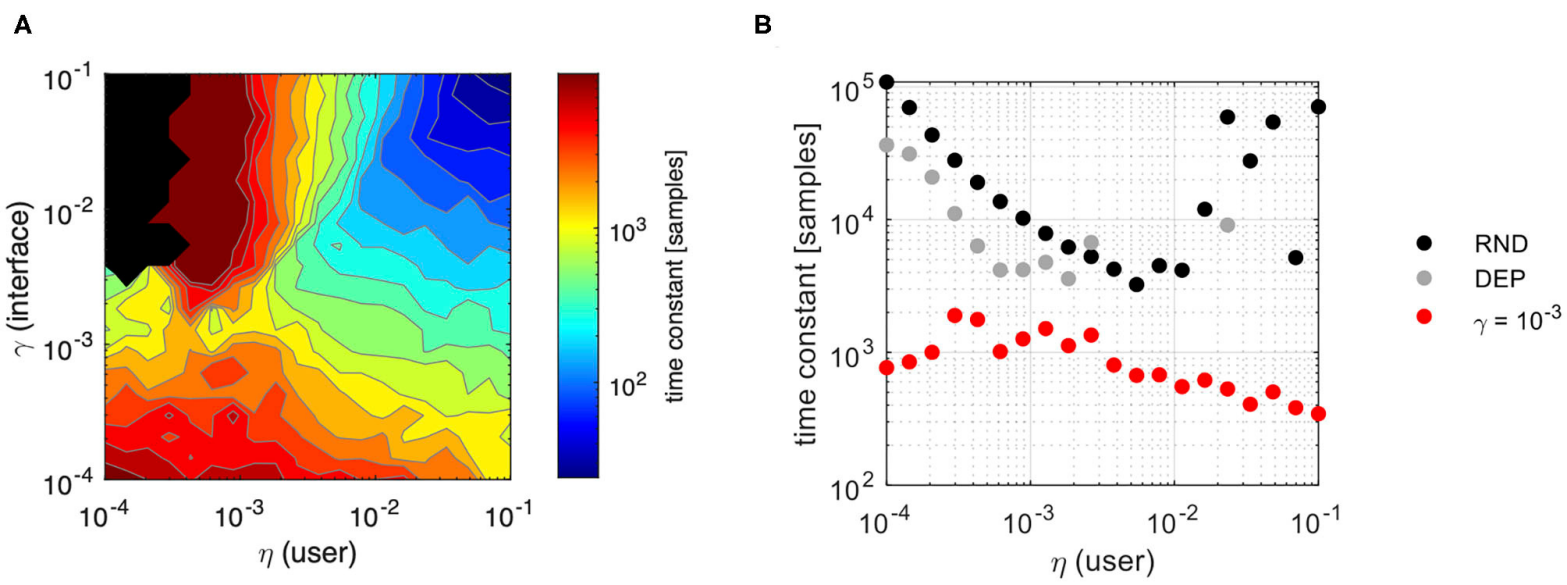
Convergence analysis. Estimated convergence rate from exponential fitting of Subspace Angle values over iterations for different combinations of user and interface rates of adaptation; **(A)** estimated time constant to convergence for Subspace Angle during co-adaptation. Only values for which the goodness of fit *R*^2^ ≥ 0.40 have been included (black regions correspond to missing data); **(B)** comparison between the estimated time constants for different degrees of user learning in the case of a static interface and independent samples (RND—black line) or dependent samples (DEP—gray line) and after interface coadaptation with γ = 10^−3^ (red line). Again, only values for which *R*^2^ ≥ 0.40 have been reported.

Finally, Figure 8 summarizes the effect of co-adaptation on the speed of convergence of the user-interface dyad compared to a user interacting with static interface. Panel A highlights the combined effect of the user and the interface in determining the time constant for convergence to a negotiated solution in the co-adaptation scenario. Fastest convergence is achieved when the interface adapts as fast as the user does. Interestingly, the figure suggests that interface adaptation values in the interval 0.0006 < γ < 0.0026 can yield to a reasonably fast convergence even in the case of very slow learners. Indeed, Figure 8—panel B clearly shows that interface adaptation allows reducing time to convergence compared to a static map condition consistently over the whole range of user learning rates.

Taken together, these systematic results provide an empirical evidence that co-adaptive interfaces can guide the user toward discovering more efficient solutions to the interface control problem.

## Discussion

This work introduced a mathematical framework for studying co-adaptation in body-machine interfaces that emphasizes the role of user's learning in shaping the interaction with an adaptive interface. The framework formulates co-adaptation in a task-independent and model-free way assuming that the user and the interface co-adapt toward maximizing control efficiency.

The generality of this novel framework can be exploited to simulate a variety of interaction scenarios, as knowledge of user intent or task goals is not required, it allows investigating the parameters leading to optimal co-adaptation dynamics and allows to empirically demonstrate the superiority of co-adaptation over user adaptation.

In the next sections, we will discuss the potential implications of our assumptions about user learning, what general recommendations for choosing the interface adaptation rate can be derived from our analysis, and finally how the framework could be generalized beyond the context of body-machine interfaces and what the possible limitations of the proposed formulation are.

### What Can a Simple User Model Tell Us?

The starting point of our reasoning lies in the search for a suitable strategy in the context of learning in redundant environments (Newell and Vaillancourt, 2001; Ranganathan et al., 2013; Pacheco et al., 2019). It has been suggested that the earliest stages of learning a new skill are primarily reliant on mechanisms of exploration and reinforcement (Bernardi et al., 2015), whereas model-based learning requires a pre-existing internal representation of the environment the user is interacting with (Huang et al., 2011). When a first-time user is faced with the problem of controlling an external object through an interface, the need to attain task goals is blurred by the need to identify a suitable way to transfer intended commands to the object, in other words, to discover the causality of the interface as a “tool” (Maravita and Iriki, 2004).

Hence, we postulated a division of roles between the half of the user that aims at optimizing task performance and the half that aims to build a sensorimotor representation of the interface (Di Pino et al., 2014; Bernardi et al., 2015). Here we addressed how to model the second problem, reducing the first problem to its observable consequences. In particular, instead of modeling the process of generating reaching movements toward a target in the task space, we resolved to reproducing the observable traces of a reaching command—a trajectory of statistically dependent points. Then, we assumed that the problem of learning to interact with the interface successfully—that does not imply optimally or efficiently—could be solved by a model-free mechanism relying on reinforcement of successful actions through a process of trial and error (Huang et al., 2011; Sutton and Barto, 2018). The process results in the consolidation of memories through use-dependent plasticity (Krutky and Perreault, 2005; Diedrichsen et al., 2010). We are aware that this interpretation is fairly simplified, as multiple model-free and model-based mechanisms are likely contributing jointly to skill acquisition (Dingwell et al., 2002; Pierella et al., 2019). Nevertheless, we asked whether this simplified vision could be sufficient to reproduce features of skill learning expressed when interacting for the first time with a redundant tool in the form of a body-machine interface.

The results of our simulations suggest that this simplified model is indeed able to faithfully represent the emergence of a stable subspace of actions that result from the interaction with the interface. Interestingly, our model was also able to reproduce another feature of learning, that is the emergence of individual strategies as a function of the trajectory of actions generated during learning (Pacheco et al., 2019). When the model was trained on experimentally observed sequences of data, it exhibited a similar trajectory as the individual the data was produced from. Variability in model solutions across multiple simulation runs was a direct consequence of assuming that the user explores the available action space through a sequence of dependent observations. Variability became irrelevant when the simulated user was given the possibility to sample the action space through independent observations. Moreover, learning with dependent action sequences made the model more likely to converge to sub-optimal solutions from the point of view of control efficiency, a tendency also observed in practice (De Santis and Mussa-Ivaldi, 2020). This result has important practical implications for the design and interpretation of studies involving sensorimotor learning, as it highlights how the simple choice of target locations in the workspace may implicitly bias the subject's behavior (Rohde et al., 2019).

### How to Choose the Learning Rate for Optimal Co-adaptation?

Before answering, we should first ponder another question, that is “what should be considered optimal in co-adaptation?” For some, co-adaptation was successful if it could lead to improving control performance in a specific task (Orsborn et al., 2012; Hahne et al., 2015; Abu-Rmileh et al., 2019). For others, optimal co-adaptation was able to return performance to the baseline level after compensating for interface instabilities (Jarosiewicz et al., 2015; Kao et al., 2017). Here, we suggest that co-adaptation is optimal if it maximizes control efficiency. In this sense, the unsupervised paradigm for interface adaptation proposed here, was successfully applied in two studies, the first implementing a linear interface as described in this work (De Santis et al., 2018) and the second using a non-linear interface implemented through an autoencoder network (Rizzoglio et al., 2021). In the latter, co-adaptation led to both an increase in control efficiency and an improvement in performance during a reaching task.

Our simulations predict that for a same user (i.e., a model characterized by a certain learning rate and initial condition) co-adaptation leads to greater control efficiency than what the user would have otherwise attained and allows reaching a stable equilibrium faster. We found that, for a given user learning rate, interface efficiency could be maximized for a relatively broad range of interface adaptation rates. However, choosing an adaptation rate similar to the time scale of user learning would lead to the best performance, both in terms of steady state solution and in terms of stability. This result seems to disagree with that of Igual et al. (2019), where imbalanced learning rates between the adaptive myoelectric controller and the user were found more likely to drive convergence to a stable equilibrium in a reaching task. Simulation results within our framework also suggest that interface adaptation should be chosen conservatively small rather than too large. In fact, adaptation rates that are smaller than the user learning rate still lead to improvements in control efficiency at the cost of a slightly slower convergence and possibly to suboptimal solutions, while larger learning rates tend to introduce instability in the solution and inhibit joint adaptation, in agreement with the results of Hahne et al. (2015) and Müller et al. (2017). From the simulations carried out here, a value of interface adaptation rate close to 10^−3^ seems to be the recommended conservative choice. Indeed, this value is close to the empirical choice for the adaptation rate of the interface (η = 0.002) tested in De Santis et al. (2018), and the value of 0.005 identified as optimal in the tests performed in Hahne et al. (2015).

One point should be stressed. Interface co-adaptation is a viable way to optimize interface control, but it does not eliminate the need for user learning. Plug-and-play interfaces (Silversmith et al., 2020) are only applicable whenever a stable action subspace (or neural manifold) for a certain task has formed through repeated exposure to the interface.

### Framework Generalizability and Limitations

One of the main features of the proposed framework is that it allows framing co-adaptation in a context that is task independent. However, we believe this should not hinder its application to instances when task goals and dynamics are well-known and/or can be modeled. In fact, the user generative model described here could be replaced with a model that select actions in a task-dependent or goal-oriented way. It could potentially be further expanded to account for the role of error-based and/or model-based mechanisms in determining the sequence of actions and corrections the user produces in response to the feedback from the interface and the task goals or constraints.

In this way, the framework could be exploited to study the effect of co-adaptation in the interaction between the user and the interface in specific tasks, as for instance during reaching, or in response to other design factors, such as the position and sequence of the reaching targets.

The other distinctive trait that increases the applicability of the framework is that interface adaptation in unsupervised, does not require any optimization routine, and can be run in real-time. The proposed interface is particularly well-suited for applications that make use of the statistics of the user's input to encode a lower dimensional space in which movement of the external device occurs, such as body-machine interfaces. However, supervised approaches are far more popular in brain computer interfaces where the decoder is trained to recognize motor intention. We believe that these two formulations are not incompatible, rather they can take advantage of each other's strengths. Stable decoders rely on the existence of consolidated patterns of brain activity, often referred to as neural manifolds (Gallego et al., 2017, 2020). Unsupervised adaptive approaches for subspace estimation in non-stationary situations could be applied to identify emergent patterns of brain activity concurrent to interface use upon which the decoder could be built. Degenhart and colleagues proposed a very similar concept to stabilize a brain-machine interface across days, with the difference that their approach relied on the existence of an already consolidated neural manifold (Degenhart et al., 2020).

One possible limitation to the proposed framework is that the analysis has been carried out for a user interacting with a linear interface, whose representational power may be limited when the input distribution presents considerable non-linearities (Portnova-Fahreeva et al., 2020). In a recent work (Rizzoglio et al., 2021) we proposed an implementation of a co-adaptive interface that makes use of an iteratively trained autoencoder network (Kramer, 1991) to perform unsupervised dimensionality reduction as opposed to standard principal components analysis. Hence, we believe the framework could be easily generalized to implement non-linear dimensionality reduction for manifold estimation and future work should investigate whether the conclusions drawn here still apply to non-linear interfaces.

A second limitation is that our approach does not allow considering the effects of interface adaptation on the explicit components of motor planning and on the engagement of model-based mechanisms in response to altered feedback. It is indeed possible that changes in the interface map introduce variability in the sensory feedback and further inconsistencies that negatively affect the performance in the task, triggering other mechanisms of adaptation (e.g., error-based). This phenomenon may be amplified whenever the learning rate imbalance triggers instability in the interface map. For an appropriate choice of learning rates, this effect is expected to rapidly disappear as soon as the system reaches an equilibrium.

Finally, as we have focused our investigation on the impact of co-adaptation on the convergence and stability of the system in the initial phases of learning, we have not specifically addressed the problem of stability over a long period of time. Nevertheless, the results from simulations foster the idea that the joint system reaches a point of equilibrium, suggesting that the solution could be stable over extended interaction despite the adaptive model having a constant learning rate. It is however possible that non-stationary adaptation rates for the interface may lead to further stability enhancement.

## Data Availability Statement

The data analyzed in this study is subject to the following licenses/restrictions: The code and the datasets generated and analyzed for this study are available from the corresponding author on reasonable request. Requests to access these datasets should be directed to Dalia De Santis, dalia.desantis@gmail.com.

## Author Contributions

DD conceived the work, formulated the model, carried out model simulations, data analysis and interpretation, drafted the manuscript, revised, and approved the submitted version.

## Conflict of Interest

The author declares that the research was conducted in the absence of any commercial or financial relationships that could be constructed as a potential conflict of interest.
